# Antioxidant Effects of Caffeic Acid Lead to Protection of *Drosophila* Intestinal Stem Cell Aging

**DOI:** 10.3389/fcell.2021.735483

**Published:** 2021-09-09

**Authors:** Xiao Sheng, Yuedan Zhu, Juanyu Zhou, La Yan, Gang Du, Zhiming Liu, Haiyang Chen

**Affiliations:** ^1^Key Laboratory of Gene Engineering of the Ministry of Education, State Key Laboratory of Biocontrol, School of Life Sciences, Sun Yat-sen University, Guangzhou, China; ^2^Laboratory of Metabolism and Aging Research, National Clinical Research Center for Geriatrics, West China Hospital, Sichuan University, Chengdu, China

**Keywords:** caffeic acid, intestinal stem cell, gut, aging, antioxidant activity

## Abstract

The dysfunction or exhaustion of adult stem cells during aging is closely linked to tissue aging and age-related diseases. Circumventing this aging-related exhaustion of adult stem cells could significantly alleviate the functional decline of organs. Therefore, identifying small molecular compounds that could prevent the age-related decline of stem cell function is a primary goal in anti-aging research. Caffeic acid (CA), a phenolic compound synthesized in plants, offers substantial health benefits for multiple age-related diseases and aging. However, the effects of CA on adult stem cells remain largely unknown. Using the *Drosophila* midgut as a model, this study showed that oral administration with CA significantly delayed age-associated *Drosophila* gut dysplasia caused by the dysregulation of intestinal stem cells (ISCs) upon aging. Moreover, administering CA retarded the decline of intestinal functions in aged *Drosophila* and prevented hyperproliferation of age-associated ISC by suppressing oxidative stress-associated JNK signaling. On the other hand, CA supplementation significantly ameliorated the gut hyperplasia defect and reduced environmentally induced mortality, revealing the positive effects of CA on tolerance to stress responses. Taken together, our findings report a crucial role of CA in delaying age-related changes in ISCs of *Drosophila*.

## Introduction

Adult stem cells play an important role in tissue homeostasis and regeneration through their self-renewal and differentiation ability (Micchelli and Perrimon, 2006; Ohlstein and Spradling, 2006, 2007; Jiang and Edgar, 2011). Age-related dysregulation of adult stem cells is closely associated with tissue aging and age-related diseases, such as cancer and degenerative diseases (Rando, 2006; Apidianakis and Rahme, 2011; Cable et al., 2020). Although stem cells could be considered immortal, their proliferative and differentiation abilities are characterized by an age-associated decline (Schultz and Sinclair, 2016). Thus, stem cell exhaustion has been considered to be one of the most critical hallmarks of organismal aging. Elsewhere, preventing stem cell exhaustion has been developed as a promising strategy for anti-aging research (López-Otín et al., 2013). Nutritional control is emerging as an important mechanism for preventing the functional decline of adult stem cells (Mana et al., 2017). Apart from caloric restriction, some drugs and compounds have been shown to promote longevity and delay the onset of age-related diseases by regulating stem cell self-renewal and differentiation (Du et al., 2020, 2021). Besides, many natural compounds have drawn attention to anti-aging effects (Pan et al., 2012; Ding et al., 2017; Corrêa et al., 2018). However, the relations between these compounds and stem cell functions need to be explored further.

Caffeic acid (CA) is an endogenous phenolic phytochemical widely found in diets (Olthof et al., 2001; Scalbert et al., 2002; Thomsen et al., 2018). Like flavonoid constituents and other phenolic acids, CA and its derivatives are important ingredients with potent anti-cancer activity. The main suggested mechanisms for the anti-cancer activity of CA are antioxidant, antiinflammatory, and antiproliferative, which have been reported in several cancer types or cancer cell lines, such as human small cell lung cancer, hepatocarcinoma, melanomas, prostate cancer, and cervical cancer (Ozturk et al., 2012; Tyszka-Czochara et al., 2018; Espíndola et al., 2019; Fırat et al., 2019; Mo et al., 2020). In recent years, accumulating evidence has demonstrated the beneficial outcomes of CA against multiple diseases, especially age-related diseases, such as Parkinson’s disease, Alzheimer’s, cardiovascular disease, and kidney disease (Eşrefoğlu et al., 2012; Fukuda et al., 2015; Habtemariam, 2017; Chang et al., 2019; Zhang et al., 2019a). Besides, CA is also considered as a promising anti-aging intervention for extending lifespan and preventing aging-associated disorders in various organisms and animal models by a systematic pharmacological approach, including *Mus musculus*, *Saccharomyces cerevisiae*, *Caenorhabditis elegans*, and *Drosophila melanogaster* (Pietsch et al., 2011; Fang et al., 2017; Li et al., 2020). However, the effects of CA on age-related functional decline in adult stem cells remain unexplored.

**FIGURE 1:**
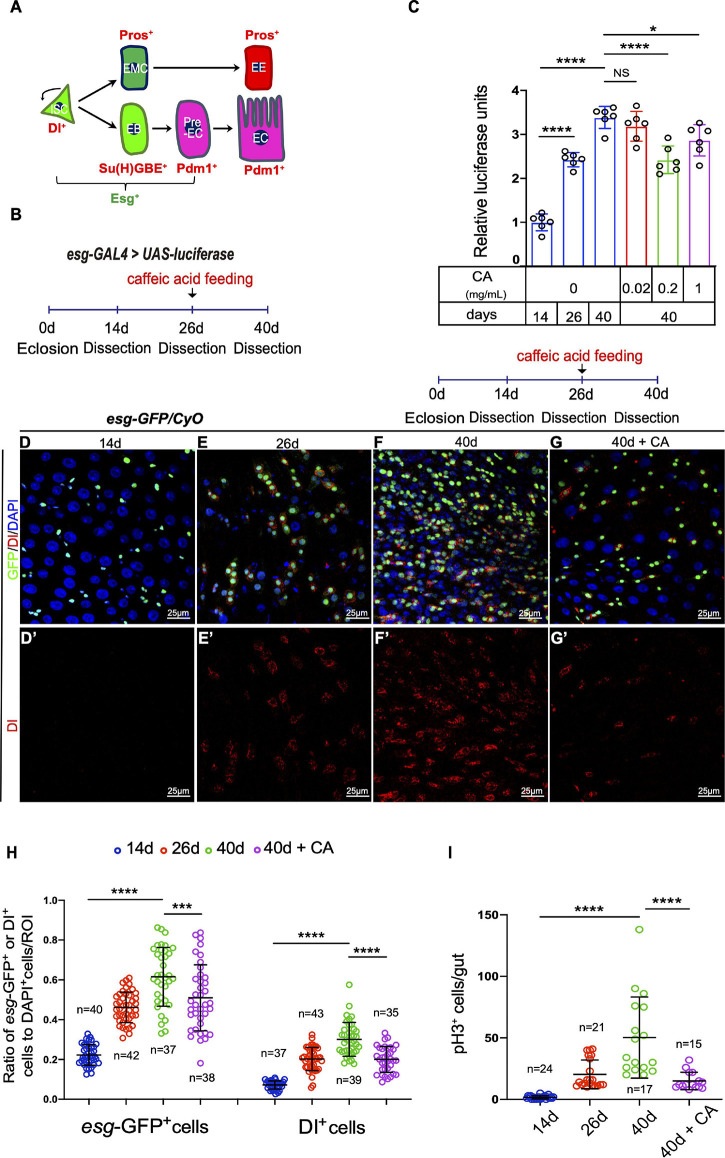
Caffeic acid (CA) supplementation prevents the ISC hyperproliferation in aged *Drosophila*. **(A)** Cartoon model of *Drosophila* ISC lineages. An ISC (Dl^+^ and Esg^+^) divides asymmetrically to a diploid precursor enteroblast [EB; Esg^+^ and Su(H)GBE^+^] or a diploid precursor enteroendocrine mother cell (EMC). Notch activated EBs (pre-EC; Esg^+^ and Pdm1^+^) further differentiate into enterocytes (ECs; Pdm1^+^), whereas enteroendocrine cells (EEs; Pros^+^) are derived from ISCs in a Notch-independent manner *via* a diploid precursor enteroendocrine mother cell (EMC) intermediate. **(B)** An illustration of the *Drosophila* “*esg* > luciferase” reporter system. *Drosophila* with luciferase expression driven by *esg*-GAL4 was treated with CA on the 26th day after eclosion. After administration for 14 days, the midguts were dissected, and the luciferase activity was measured. **(C)** Quantification of the luciferase activity of the midguts of flies at 14, 26, and 40 days without CA supplementation (orderly marked with three blue columns) and 40-day flies with three different concentrations of CA (orderly marked with red, green, and purple columns). Error bars represent the standard deviation (SD) of six independent experiments. **(D–G)** Immunofluorescence images of the R4 region of dissected midguts of 14-day *Drosophila* **(D)**, 26-day *Drosophila* **(E)**, 40-day *Drosophila* **(F)**, and 40-day *Drosophila* with 0.2 mg/mL CA supplementation **(G)**. *esg*-GFP (green; ISC and EB markers) and Dl (red; ISC marker). Panels **(D–G)** represent merged images and panels **(D’–G’)** are for Dl (red) only. **(H,I)** Quantification of the number of *esg*-GFP^+^, Dl^+^, and pH3^+^ cells in experiments **(D–G)**. N (from left to right) = 40, 42, 37, 38, 37, 43, 39, and 35 in panel **(H)**. N (from left to right) = 24, 21, 17, and 15 in panel **(I)**. DAPI stained nuclei are shown in blue in panels **(D–G)**. Scale bars represent 25 μm in panels **(D–G)**. Error bars represent SDs. One-way ANOVA test, *P < 0.05, ***P < 0.001, ****P < 0.0001, and non-significant (NS) represents *P* > 0.05.

Due to its easy genetic manipulation and well-defined stem cell lineage, the *Drosophila* midgut has been a prime paradigm for studying stem cell functions during adult tissue homeostasis and aging. *Drosophila* intestinal stem cells (ISCs) are a cell population located in the basal membrane of the digestive tract (Miguel-Aliaga et al., 2018). They could divide to self-renew and produce either enteroblasts (EBs) or enteroendocrine mother cells (EMCs) depending on Notch activity. Subsequently, EBs with high Notch signaling levels further differentiate into absorptive enterocytes (ECs), and EMCs produce secretory enteroendocrine cells (EEs). Different cell types of *Drosophila* midguts could be distinguished by examining the cell-specific markers (Figure 1A). Consistent with previous studies, the number of ISCs and progenitor cells rise dramatically with age (Biteau et al., 2008; Choi et al., 2008). Several signaling pathways have been reported to function in these age-related changes in ISCs, such as c-Jun N-terminal kinase (JNK) signaling pathway (Biteau et al., 2008), mTOR signaling pathway (Johnson et al., 2013), and insulin signaling (Biteau et al., 2010; Kao et al., 2015). Also, several factors have been shown their roles in modulating the age-related changes in ISCs, such as PVF2, Sox21a, and GATAe (Choi et al., 2008; Wu et al., 2021). Additionally, preventing ISC hyperproliferation (either by genetic manipulation or drug administration) has been shown to extend the lifespan of *Drosophila* (Gervais and Bardin, 2017). The above results of these previous studies strongly suggest that the *Drosophila* midgut is an ideal model to study the effect of CA in regulating stem cell aging.

In this study, we report that CA supplementation could attenuate the age-associated hyperproliferation of *Drosophila* ISCs by suppressing oxidative stress-associated JNK signaling.

## Results

### Caffeic Acid Supplementation Prevents Gut Hyperplasia in Aged *Drosophila*

In aged *Drosophila*, the ISCs and progenitor cells of the midguts increased sharply as indicated by the accumulation of *escargot* (*esg*)-positive (*esg*^+^) cells (ISCs and progenitor cells). Meanwhile, the proliferation rate of the ISCs increased drastically, leading to an accumulation of Phospho-Histone 3-positive (pH3^+^) cells (mitotic cells; intestinal epithelia ISCs are the only cells in *Drosophila* midgut with proliferation ability) (Choi et al., 2008; Cui et al., 2019). To test whether CA exerts beneficial effects on preventing ISC aging in *Drosophila* midguts, we used an “*esg*-luciferase” reporter system to trace and quantify the real-time changes of ISCs and their differentiating progenies (Figure 1B). Through this system, we found that CA supplementation showed obvious delayed effects on *esg*^+^ cell accumulation in aged (40 days) *Drosophila* midguts (Figure 1C). Among the three tested concentrations (0.02, 0.2, and 1 mg/mL), 0.2 mg/mL CA supplementation was the optimal concentration of delaying *esg*^+^ cell accumulation in aged *Drosophila* (Figure 1C). To further explore the role of CA in preventing ISC hyperproliferation, an *esg*-GFP reporter line was used to make ISCs and their differentiating progenies in aged midguts visualization. These ISCs were marked by the Delta (Dl) antibody staining. Upon aging, the number of *esg*-GFP^+^, Dl^+^, and pH3^+^ cells continuously increased in *Drosophila* midguts (Figures 1D–F,H,I), which is consistent with previous studies (Choi et al., 2008; Cui et al., 2019). This study showed that the accumulation of *esg*-GFP^+^ and Dl^+^ cells were significantly decreased in CA pre-treated (starting at the 26th day after eclosion) *Drosophila* than aged flies without CA supplementation (Figures 1F–H). According to the mitotic rate indicated by pH3 staining, CA supplementation prevented aging-associated ISC hyperproliferation in *Drosophila* (Figure 1I). These results indicate that CA restrained ISC aging in *Drosophila*.

### Administration of CA Prevents the Age-Related Decline of Intestinal Functions

**FIGURE 2:**
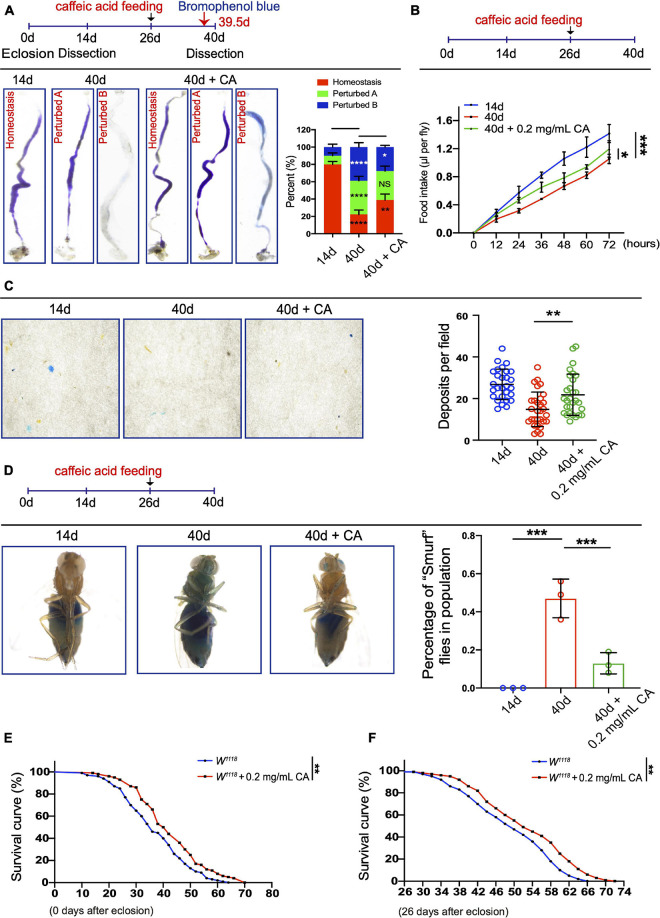
Inhibitory effects of CA on the age-related decline in intestinal functions. **(A)** Typical images (left) and quantification (right) of GI tract of *Drosophila* fed with the pH indicator Bromophenol Blue. There are three types of the GI tract of flies fed with Bromophenol blue, including “Homeostasis,” “Perturbed A,” and “Perturbed B.” *N* = 90 flies per group. The *P*-values between 14-day and 40-day are highlighted in the 40-day column. The *P*-values between 40-day and 40-day with 0.2 mg/mL CA supplementation are highlighted in the 40-day with 0.2 mg/mL CA column. Error bars represent the SD of three independent experiments. **(B)** CAFE assay was used to measure food intake in 14- (the blue line), 40- (the red line), and 40-day flies in response to 0.2 mg/mL CA supplementation (the green line). Error bars represent the SD of three independent experiments. **(C)** Representative images (left) and quantification (right) of excretion from flies fed with Bromophenol Blue. Excretions are quantified for 28, 30, and 29 fields in each group of 12 flies. 28, 30, and 29 represent the field and the deposits were counted per field. Error bars represent the SD of three independent experiments. **(D)** Representative images (left) and quantification (right) of the percentage of “Smurf” flies of 14-, 40-, and 40-day with 0.2 mg/mL CA supplementation after consuming a non-absorbed food dye. Error bars represent the SD of three independent experiments. **(E)** Life span assay. Survival curve (%) of female *W*^1118^ flies treated with (marked with the red curve) or without (marked with the blue curve) 0.2 mg/mL CA starting from the day after eclosion. *N* = 100 flies per group. Three independent experiments were performed. **(F)** Life span assay. Survival curve (%) of female *W*^1118^ flies treated with (marked with the red curve) or without (marked with the blue curve) 0.2 mg/mL CA starting from the 26th-day post-eclosion. *N* = 100 flies per group. Three independent experiments were repeated. Error bars represent SDs. One-way ANOVA test, Log-rank test for panels **(E,F)**. *P < 0.05, **P < 0.01, ***P < 0.001, ****P < 0.0001, and non-significant (NS) represents *P* > 0.05.

Age-associated ISC hyperproliferation results in a remarkable decline in midgut digestive functions, which is characterized by the loss of gastrointestinal acid-base homeostasis and the decline of food intake and excretion (Cognigni et al., 2011; Deshpande et al., 2014; Li et al., 2016). Since CA supplementation can prevent age-related ISC over-proliferation, we sought to determine whether it also retards the decline of intestinal functions in aged *Drosophila*. In aged *Drosophila*, 0.2 mg/mL CA supplementation showed the obviously retardant effects toward the deterioration of gastrointestinal acid-base homeostasis compared with the other doses (Figure 2A and Supplementary Figure 1A). Moreover, 0.2 mg/mL CA administration partially mitigated the decrease of food intake (Figure 2B and Supplementary Figure 1B) and excretion (Figure 2C and Supplementary Figure 1C) in aged *Drosophila*. The physical barrier integrity of gut tissue is important for intestinal homeostasis and digestive functions. It has been reported that the physical barrier integrity of the gut gradually wears out upon aging (Rera et al., 2012). Thus, we conducted the “Smurf” assay to test the effects of CA on intestinal barrier integrity (Rera et al., 2012). 0.2 mg/mL CA strengthened the gut barrier function of *Drosophila* upon aging (Figure 2D and Supplementary Figure 1D). Furthermore, lifespan analyses indicated that 0.2 mg/mL CA supplementation extended the *Drosophila* lifespan for 6 days than control (Figure 2E), which is consistent with a previous study (Li et al., 2020). Moreover, we found that flies with CA fed from the mid-aged (26-day old) could also prolong their lifespan for 6 days than control (Figure 2F). While, supplementation with 0.02 mg/mL CA had no effect on the lifespan of flies (Supplementary Figure 1E).

### Caffeic Acid Supplementation Prevents the Environmental Stimuli-Induced Gut Hyperplasia and Improves Stress Tolerance in *Drosophila*

**FIGURE 3:**
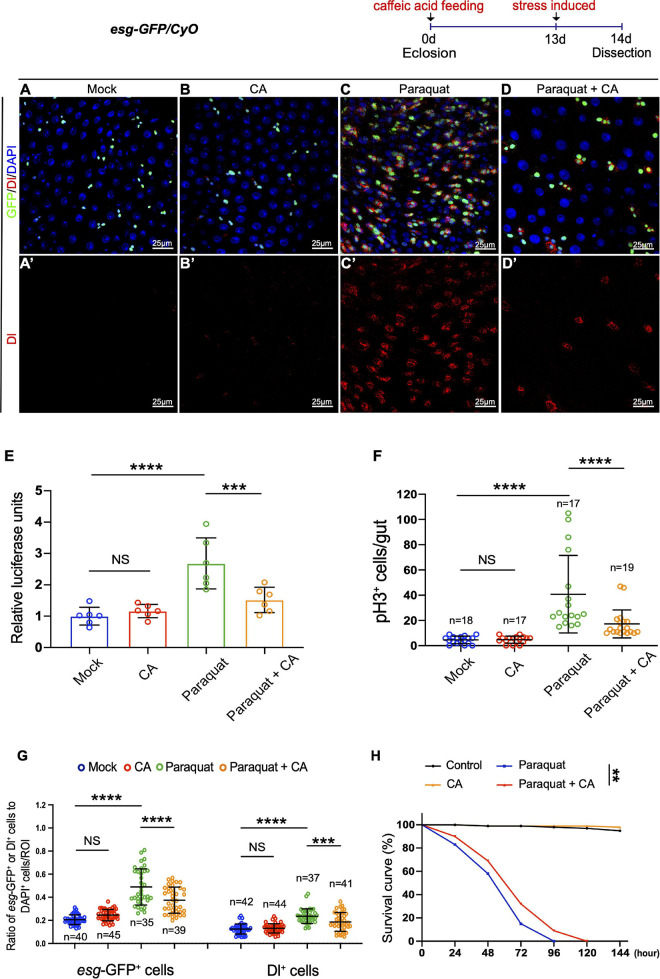
Caffeic acid (CA) supplementation prevents stress-induced ISC hyperproliferation and improves stress tolerance in *Drosophila*. **(A–D)** Immunofluorescence images of the R4 region of dissected midguts of 14-day *Drosophila*. **(A)** Mock represents the control. **(B)** Caffeic acid treatment. **(C)** Paraquat. **(D)** Paraquat with CA supplementation. *esg-*GFP (green; ISC, and EB markers) and Dl (red; ISC marker). Panels **(A–D)** represent merged images and panels **(A’–D’)** are for Dl (red) only. **(E)** Quantification of the luciferase activity of midguts of Mock, CA, Paraquat, and Paraquat with CA treated flies. Error bars represent the SD of six independent experiments. **(F)** Quantification of the number of pH3^+^ cells in experiments **(A–D)**. N (from left to right) = 18, 17, 17, and 19. **(G)** Quantification of the number of *esg*-GFP^+^ and Dl^+^ cells in experiments **(A–D)**. N (from left to right) = 40, 45, 35, 39, 42, 44, 37, and 41. **(H)** Survival percentage of flies with (marked with the red curve) and without (marked with the blue curve) CA supplementation under paraquat treatment. *N* = 100 flies per group. Three independent experiments were repeated. DAPI stained nuclei are shown in blue in panels **(A–D)**. Scale bars represent 25 μm in panels **(A–D)**. Error bars represent SDs. One-way ANOVA test, Log-rank test for panel **(H)**. **P < 0.01, ***P < 0.001, ****P < 0.0001, and non-significant (NS) represents *P* > 0.05.

Accumulation of environmental stress-induced damages is one of the factors causing organismal aging (Lavretsky and Newhouse, 2012; Luo et al., 2020). Paraquat (PQ; 1,1′-dimethyl-4,4′-bipyridinium dichloride), a non-selective ammonium herbicide, is widely used in agriculture (Brian et al., 1958). When administered *in vivo*, PQ undergoes NADPH-dependent reduction, generating a stable paraquat radical, which reacts with oxygen to generate superoxide anion (Bus and Gibson, 1984). Excess superoxide anion radicals could cause lipid peroxidation, protein carbonylation, oxidation of protein thiols, and DNA damage, which are toxic to mammals and are involved in cell death and aging (Guan et al., 2017). Caffeic acid has been reported to exhibit various pharmacological properties, including antioxidant, anti-inflammatory, and immunomodulatory activities (Olthof et al., 2001; Bezerra et al., 2012). Here, we designed an experiment to examine the protective effects of CA against acute oxidative stress induced by PQ in *Drosophila*. Young *Drosophila* midguts exposed to oxidative challenges caused by PQ exhibited ISC hyperproliferation (Figure 3C), similar to aged *Drosophila* (Figure 1F). However, CA supplementation significantly ameliorated the gut hyperplasia defect induced by PQ treatment (Figures 3A–G).

Subsequently, we found that supplementing 0.2 mg/mL CA increased the survival rate of flies under constant exposure (induced by continuously feeding with PQ) than the control group (Figure 3H). Thus, stress response revealed the positive effects of CA on tolerance to environmental stimuli.

### Caffeic Acid Supplementation Prevents ISC Aging by Promoting Oxidation Resistance

**FIGURE 4:**
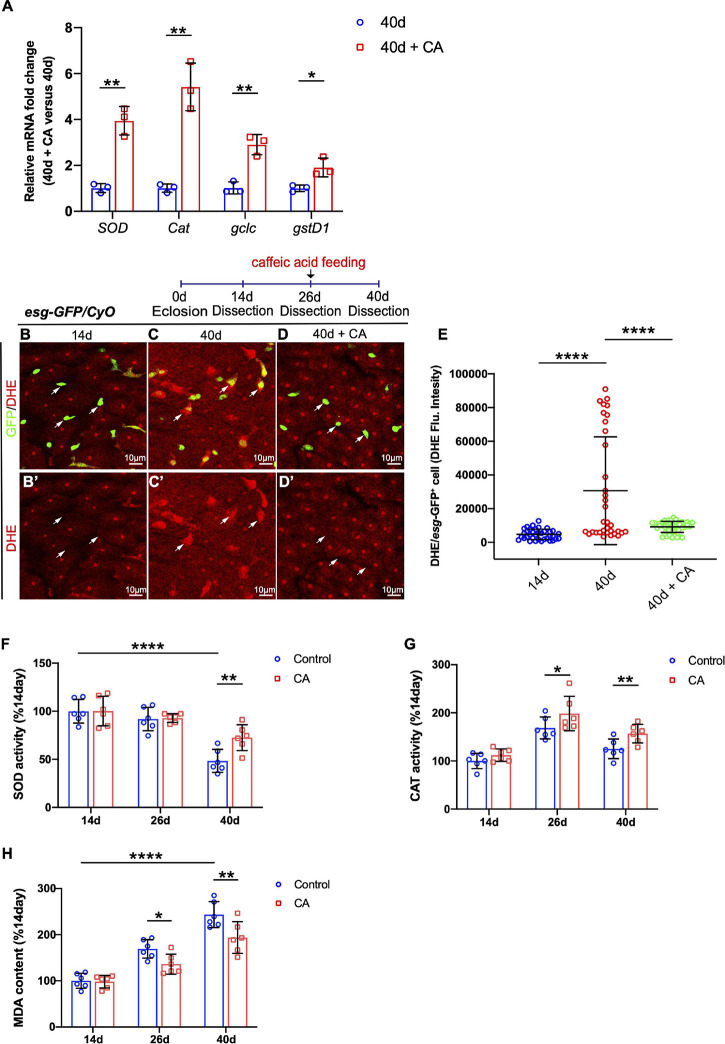
Caffeic acid (CA) supplementation prevents ISC aging by promoting oxidation resistance. **(A)** Quantitative real-time PCR (RT–qPCR) detection of the mRNA expression of *SOD*, *Cat*, *gclc*, and *gstD1* in the midguts of aged flies with or without CA supplementation. Error bars represent the SD of three independent experiments. **(B–D)** Representative immunofluorescence images of the R4 region of dissected midguts of 14-, 40-, and 40-day flies with CA supplementation. Arrows point to individual *esg*-positive cells. *esg-*GFP (green; ISC and EB markers) and DHE (red; an indicator of ROS). Panels **(B–D)** represent merged images and panels **(B’–D’)** are for DHE (red) only. **(E)** Quantification of DHE fluorescence in *esg*-positive cells of 14-, 40-, and 40-day flies with CA supplementation. Each dot represents one cell. **(F)** Quantification of the SOD activity (U/mg prot) of the midguts of flies fed with CA (marked with red columns) and without CA (marked with blue columns) at 14, 26, and 40 days. Error bars represent the SD of six independent experiments. **(G)** Quantification of the CAT activity (U/mg prot) of the midguts of flies fed with CA (marked with red columns) and without CA (marked with blue columns) at 14, 26, and 40 days. Error bars represent the SD of six independent experiments. **(H)** Quantification of the MDA content (nmol/mg prot) of the midguts of flies fed with CA (marked with red columns) and without CA (marked with blue columns) at 14, 26, and 40 days. Error bars represent the SD of six independent experiments. Scale bars represent 10 μm in panels **(B–D)**. Error bars represent SDs. One-way and two-way ANOVA test, *P < 0.05, **P < 0.01, ****P < 0.0001, and non-significant (NS) represents *P* > 0.05.

Since CA is an oxygen radical scavenger under *in vitro* and *in vivo* conditions (Nardini et al., 1995; Gülçin, 2006), our study hypothesized that it might restrain *Drosophila* intestinal stem cell aging through its antioxidant ability. Therefore, real-time quantitative PCR (RT–qPCR) analyses were performed. The selection of resistance-related genes was according to related previous studies (Hochmuth et al., 2011; Zou et al., 2015; Zhang et al., 2019b). Analyses of the selected genes (including *SOD*, *Cat*, *gclc*, and *gstD1*) showed that CA supplementation upregulates these oxidation resistance-related genes (Figure 4A). Dihydroethidium (DHE), a redox-sensitive dye, is used to monitor endogenous ROS levels *in vivo* (Owusu-Ansah et al., 2008). When *Drosophila* aged, increased DHE fluorescence intensity was observed throughout the intestinal epithelium (Figures 4C,E). Aged flies fed with CA showed a substantially reduced DHE activity in midguts (Figures 4B–E). Besides, we examined the effects of CA on the endogenous antioxidant enzyme activity in the antioxidant defense system, including superoxide dismutase (SOD) activity and catalase (CAT) activity (He et al., 2017). Along with aging, SOD activity was significantly decreased by 51.6% in the 40-day flies than the 14-day flies (Figure 4F). However, 40-day flies with CA supplementation distinctly increased the SOD activity by 24.2% (Figure 4F). On the other hand, the CAT activity decreased by 39.8% in the 40-day flies than the 26-day flies (Figure 4G). Nonetheless, in the CA group, CAT activity was higher than the control group in 26-day and 40-day flies (Figure 4G). Malondialdehyde (MDA) is a free radical-induced end-product of lipid peroxidation, and its content is an important marker for oxidative stress *in vivo* (Weismann et al., 2011). A significant increase of MDA content was observed in aged flies, while CA supplementation decreased the MDA content by 29.9 and 40.1% in 26-day and 40-day, respectively (Figure 4H). Thus, CA prevents gut hyperplasia in aged *Drosophila*, possibly by preventing oxidative stress.

**FIGURE 5:**
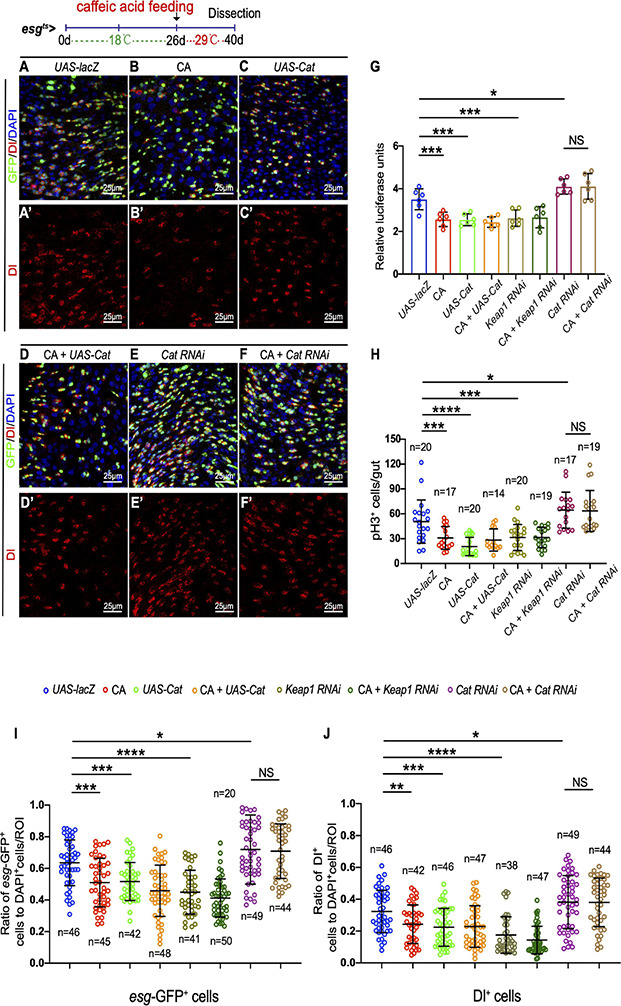
Caffeic acid (CA) prevents ISC aging *via* antioxidant ability. **(A–F)** Immunofluorescence images of the R4 region of midguts of *Drosophila* carrying *esg^*ts*^-GAL4-*driven *UAS-lacZ* (**A**, control), CA **(B)**, *UAS-Cat* **(C)**, *UAS-cat* with CA **(D)**, *Cat RNAi* **(E)**, *Cat RNAi* with CA **(F)**, *esg*-GFP (green; ISC and EB markers), and Dl (red; ISC marker). Panels **(A–F)** represent merged images and panels **(A’–F’)** are for Dl (red) only. 29°C was used to induce transgene expression (see also “Materials and Methods” section). **(G)** Quantification of the luciferase activity of *Drosophila* midguts with indicated genotypes and treatment. Error bars represent the SD of six independent experiments. **(H)** Quantification of the number of pH3^+^ cells of flies with indicated genotypes and treatment. N (from left to right) = 20, 17, 20, 14, 20, 19, 17, and 19. **(I)** Quantification of the number of *esg*-GFP^+^ cells of flies with indicated genotypes and treatment. N (from left to right) = 46, 45, 42, 48, 41, 50, 49, and 44. **(J)** Quantification of the number of Dl^+^ cells of flies with indicated genotypes and treatment. N (from left to right) = 46, 42, 46, 47, 38, 47, 49, and 44. DAPI stained nuclei are shown in blue in panels **(A–F)**. Scale bars represent 25 μm in panels **(A–F)**. Error bars represent SDs. One-way ANOVA test, *P < 0.05, **P < 0.01, ***P < 0.001, ****P < 0.0001, and non-significant (NS) represents *P* > 0.05.

Consequently, we further tested whether CA prevents the age-related functional decline in ISCs by scavenging ROS. Overexpression of *Catalase* or depletion of *Keap1* was used to eliminate the increase of ROS in aged ISCs. Catalase is a key enzyme of the antioxidant defense system in cells, catalyzing the decomposition of hydrogen peroxide into water and molecular oxygen (Glorieux and Calderon, 2017). Keap1 is a Cul3-ubiquitin ligase complex adaptor that promotes Nrf2 degradation (Fourquet et al., 2010). Consistent with previous reports (Hochmuth et al., 2011), the increase of *esg*-GFP^+^ cells, Dl^+^ cells, and pH3^+^cells was significantly inhibited in aged *Drosophila* by either overexpressing *Cat* or depleting *Keap1* (Figures 5A–C,G–J and Supplementary Figure 2A). Administering CA did not further reduce the numbers of *esg*-GFP^+^, Dl^+^, and pH3^+^ cells in aged *esg*^*ts*^-*GAL4*-driven midguts either by CAT overexpression or Keap1 depletion (Figures 5C,D,G–J and Supplementary Figures 2A,B). Furthermore, we found that CA supplementation did not rescue the increase of *esg*-GFP^+^, Dl^+^, and pH3^+^ cells triggered by *Cat* depletion in aged *Drosophila* (Figures 5E,F,G–J). Hence, ROS functions downstream of CA in preventing ISC hyperproliferation in aged *Drosophila*.

### Caffeic Acid Restrains ISC Overproliferation by Suppressing Oxidative Stress-Associated JNK Signaling

**FIGURE 6:**
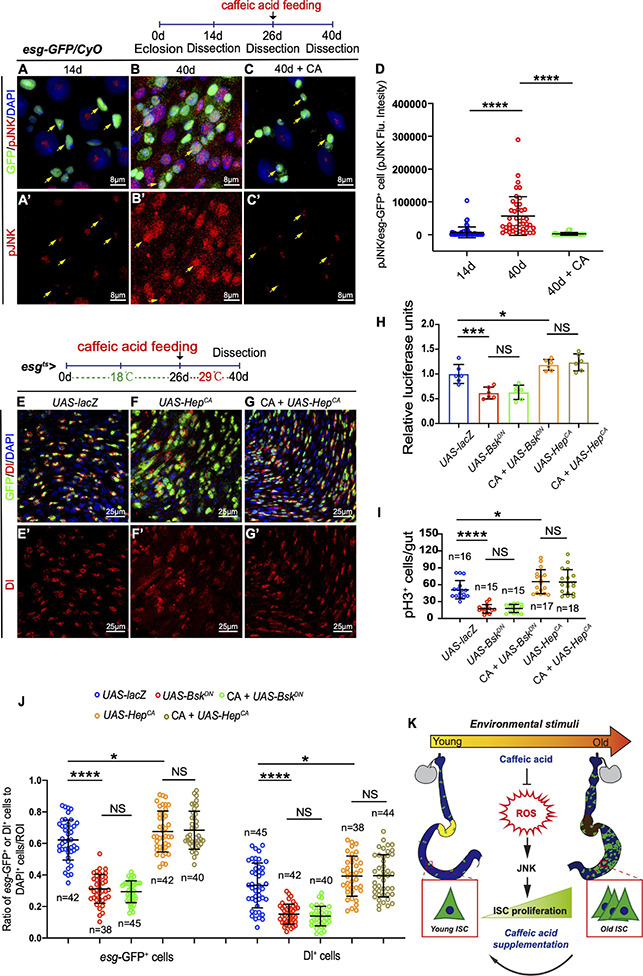
CA prevents ISC aging by counteracting oxidative stress-associated JNK activity. **(A–C)** Representative immunofluorescence images of the R4 region of dissected midguts of 14-, 40-, and 40-day flies treated with CA. Arrows indicate *esg*-positive cells. *esg*-GFP (green; ISC and EB markers) and pJNK (red; an indicator of JNK signaling activation). Panels **(A–C)** represent merged images and panels **(A’–C’)** are for pJNK (red) only. **(D)** Quantitation of the pJNK intensity in *esg*-positive cells from 14-, 40-, and 40-day flies treated with CA. Each dot corresponds to one cell. **(E–G)** Immunofluorescence images of the R4 region of dissected midguts of aged flies with indicated genotypes and treatment. *esg*-GFP (green; ISC, and EB markers) and Dl (red; ISC marker). Panels **(E–G)** represent merged images and panels **(E’–G’)** are for Dl (red) only. **(H)** Quantification of the luciferase activity of *Drosophila* midguts with indicated genotypes and treatment. Error bars represent the SD of six independent experiments. **(I)** Quantification of the numbers of pH3^+^ cells of flies with indicated genotypes and treatment. N (from left to right) = 16, 15, 15, 17, and 18. **(J)** Quantification of the number of *esg*-GFP^+^ cells, Dl^+^ cells of flies with indicated genotypes and treatment. N (from left to right) = 42, 38, 45, 42, 40, 45, 42, 40, 38, and 44. **(K)** Schematic model of the mechanism by which CA restrains *Drosophila* intestinal stem cell aging. Caffeic acid promotes oxidation resistance, which attenuates oxidative stress, prevents the over-activation of JNK signaling, and inhibits the hyperproliferation of ISCs, thus extending the lifespan and healthspan of *Drosophila*. DAPI stained nuclei are shown in blue in panels **(A–C,E–G)**. Scale bars represent 8 μm in panels **(A–C)**. Scale bars represent 25 μm in panels **(E–G)**. Error bars represent SDs. One-way ANOVA test, *P < 0.05, ***P < 0.001, ****P < 0.0001, and non-significant (NS) represents *P* > 0.05.

In *Drosophila*, JNK signaling has been reported to protect cells from oxidative stress and extend the lifespan of adult flies (Wang et al., 2003; Biteau et al., 2011). Besides, the JNK pathway is required for intestinal epithelium renewal during bacterial infection-induced ROS/oxidative stress (Buchon et al., 2009). Therefore, JNK activation in the ISCs of *Drosophila* in response to CA supplementation was monitored. Of note, aged flies fed with CA showed reduced activation of JNK activity by monitoring pJNK expression in ISCs (Figures 6A–D). Besides, the number of *esg*-GFP^+^, Dl^+^, and pH3^+^ cells did not further reduce in aged *Drosophila* supplemented with CA when the JNK signaling pathway was inhibited by overexpressing *Bsk*^*DN*^ [a dominant-negative version of the *basket (Bsk)*; *Bsk* encodes a *Drosophila* c-Jun N-terminal kinase] (Figures 6H–J and Supplementary Figures 3A,B). Activating the JNK pathway by the expression of *Hep*^*CA*^ [an active form of hemipterous *(hep)*; *hep* encodes a *Drosophila* JNK kinase] under the control of *esg-Gal4^*ts*^* is sufficient to initiate widespread ISC proliferation in the intestinal epithelium (Figure 6F). Aged flies with CA supplementation did not rescue the increase of *es*g-GFP^+^, pH3^+^, and Dl^+^ cells induced by expressing *Hep*^*CA*^ (Figures 6E–J). Thus, JNK functions downstream of CA in regulating ISC homeostasis. These results suggest that CA restrains *Drosophila* intestinal stem cell aging by suppressing oxidative stress-associated JNK signaling.

## Discussion

Over the last decades, several natural products, such as quercetin, epicatechin, and curcumin, have been shown to possess anti-aging agents that extend lifespan and prevent aging-associated diseases in various organisms and animal models (Ding et al., 2017). Although CA has also been used to treat several aging-associated diseases, its anti-aging mechanism remains largely unknown. This study reports that CA has a restrained effect on intestine stem cell aging in *Drosophila* midguts. Oral administration of CA significantly prevents age-associated hyperproliferation in ISCs and declines of intestinal functions by suppressing oxidative stress-associated JNK signaling (Figure 6K).

Besides survivorship, healthspan, which refers to the period in which organisms live without vulnerability and/or diseases, is another primary goal of anti-aging research (Ayres, 2020). For organisms, maintaining the balance and homeostasis of tissues and organs is crucial for achieving healthspan, of which the gut is a key mediator (Regan et al., 2016). In aged *Drosophila*, the ISCs undergo an increase in proliferation rate and a decrease in differentiation efficiency, prompting a considerable rise of the intestinal stem and progenitor cells number (Biteau et al., 2008; Choi et al., 2008; Cui et al., 2019). However, CA delays ISC hyperproliferation in aged flies, thus combating aging. Apart from ISCs hyperproliferation, intestinal functions, such as gut barrier integrity, digestive functions, and gut microbe homeostasis, are closely linked with intestinal homeostasis during aging (Peterson and Artis, 2014; Sommer et al., 2017). Herein, we found that the decline of intestinal functions decreased slowly in CA-treated flies than flies without CA supplementation. Meanwhile, CA substantially enhances the resistance of flies against environment-derived constant stressors with consequent improvement in their health and survival state. These results indicate that the CA-treated *Drosophila* have a better health state than the control group of the same age. Therefore, it could be considered that CA significantly extends the lifespan and prolongs the healthspan of flies. Besides, we have discovered that CA supplementation (starting from the 26th day after eclosion) improves the lifespan of mid-aged flies (Figure 2F). This suggests that we could begin CA supplementation in middle-aged flies instead of at birth. Overall, the anti-aging and protective effects of CA deserve more attention to human health.

Reactive oxygen species (ROS) derived from extrinsic or intrinsic processes contribute to the cellular redox environment and serve as critical intracellular messengers in the human body. Excessive ROS leads to oxidative stress, resulting in a broad spectrum of pathophysiological disturbances such as aging, cancer, and necrosis (Sosa et al., 2013; Giorgi et al., 2018). Moreover, ROS plays a critical role in regulating stem cell homeostasis (Hochmuth et al., 2011). Reactive oxygen species levels increase in response to aging, accompanied by the elevated proliferation of ISCs in aged *Drosophila* (Chen et al., 2021). Thus, anti-oxidative-based interventions have emerged as a vital part of the redox system balance that regulates stem cell function in high-turnover tissues (Hochmuth et al., 2011; Morris and Jasper, 2021). Because the CA structure contains two free phenolic hydroxyls, it is a natural antioxidant. Therefore, its anti-oxidative ability could not be ignored in the process of delaying stem cell aging. Indeed, our results have demonstrated that CA prevents ISC hyperproliferation through its anti-oxidative ability (Figure 6K). However, the underlying mechanism by which CA interact with the ROS signaling pathway remains largely undetermined, and the details of how CA modulates the expression of oxidation resistance-related genes are yet to be performed. Besides, increased ROS levels have been shown to influence gastrointestinal microbial community’s biodiversity. Reciprocally, gut microbes affect ROS levels. There is also an important balance between gut microbiota and intestinal epithelial homeostasis (Jones and Neish, 2017; Ballard and Towarnicki, 2020). However, the possibility that CA delays age-associated ISC hyperproliferation *via* affecting gut microbiota is lack demonstration in this study and deserves to be taken into consideration in further investigations.

Collectively, our findings demonstrate that CA is a novel and promising anti-ISC aging compound that could delay the aging process in stem cells and improve the lifespan and healthspan of *Drosophila*. As a phenolic phytochemical, the bioactivities of CA, as well as their derivates, are most likely related to their molecular structures, which are determined by molecular weight, chemical modification, link types, and chain conformation. If the association between chemical structure, biochemical characterization, and anti-ISC aging could be confirmed and the detailed mechanisms could be further clarified in future studies, it may provide new strategies toward treating age-associated or damage-induced intestinal dysfunction in humans.

## Materials and Methods

### Contact for Reagent and Resource Sharing

Requests for further information, reagents, and resources should be directed to and will be handled by the Lead Contact, Haiyang Chen (Chenhy82@scu.edu.cn).

### *Drosophila* Stocks and Husbandry

We used the *w*^1118^ allele (BDSC 3605) as the wild-type control. All flies were maintained at 25°C on standard food (cornmeal 50 g, yeast 18.75 g, sucrose 80 g, glucose 20 g, agar 5 g, and propionic acid 30 mL combined in 1 L water) under a normal dark/light cycle. GAL4-UAS-mediated RNAi or overexpression was used in this study. The temperature-sensitive GAL4-mediated gene knockdown or overexpression was repressed at 18°C. When flies eclosion, the progeny were shifted to 29°C to induce transgene expression for indicated days. Mated females were used in all experiments unless mentioned otherwise. *Drosophila* lines used in this study are listed in Supplementary Table 1.

### Immunofluorescence and Microscopy Analyses

Midguts were immersed and dissected in cold phosphate-buffered saline (PBS) before being fixed with 4% EM-grade paraformaldehyde buffer (formula: 100 mM glutamic acid, 25 mM KCl, 20 mM MgSO_4_, 4 mM Na_2_HPO_4_, 1 mM MgCl_2_, pH 7.4) for 1 h at room temperature. The midguts were then washed 3 times for 10 min each using a wash buffer (PBS plus 0.3% Triton X-100, PBST). All samples were soaked in 0.5% BSA (in PBST buffer) for 30 min, followed by incubation with primary antibodies (dissolve in PBST buffer) at 4°C overnight. Then, the samples were incubated with secondary antibodies and DAPI for 2 h at room temperature, followed by the same washing steps above.

Primary antibodies used in immunostaining are listed in the Reagent Table (Supplementary Table 2). The secondary antibodies (Alexa 488, Alexa 568) were diluted in PBST and used at 1:2000. Nuclei were stained with 4,6-diamidino-2-phenylindole (DAPI; Sigma) at a final concentration of 1 μg/mL.

Leica TCS-SP8 confocal microscope was used to obtain all immunofluorescent images. Adobe Photoshop and Illustrator were used to assemble all the images.

### DHE Staining

Dihydroethidium (DHE) staining was used to probe the levels of ROS (Hochmuth et al., 2011). Briefly, midguts were dissected and incubated in 30 μM DHE (Invitrogen) in the dark at room temperature for 5 min. Then, they were washed 3 times with PBS, and all images were captured immediately *via* Leica TCS-SP8 confocal microscope.

### qRT-PCR Analysis

Midguts were dissected in 4°C diethyl pyrocarbonate (DEPC)-treated water-PBS. Total RNA was extracted from dissected midguts with Trizol reagent (Invitrogen). Template RNA (1 μg) was used to generate cDNA by reverse transcription. There are two steps for cDNA production (Evo M-MLV RT Kit with gDNA Clean for qPCR, AG11705). First, removal of genomic DNA:

**Table:** 

**Components**	**Quantity**
gDNA Clean Reagent	1 μL
5X gDNA Clean Buffer	2 μL
Total RNA	–
RNase free water	up to 10 μL

	
Reaction conditions:	42°C	2 min
	4°C	–

Next, reverse transcription:

**Table:** 

**Components**	**Quantity**
Reaction mixture	10 μL
Evo M-MLV RTase Enzyme Mix	1 μL
Oligo dT (18T) Primer (50 μM)	1 μL
Random 6 mers Primer (100 μM)	1 μL
5X RTase Reaction Buffer Mix I	4 μL
RNase free water	3 μL
Total	20 μL

Reaction conditions:	37°C	15 min
	85°C	5 s
	4°C	–

Then, the cDNA was used to perform quantitative RT-PCR on a QuantStudio 5 System (Thermo Fisher Scientific). The following primers were used: SOD sense, 5′-CAAGGG CACGGTTTTCTTC-3′ and antisense, 5′-CCTCACCGGAGA CCTTCAC-3′, CAT sense, 5′-TTCGATGTCACCAAGGTCTG-3′ and antisense, 5′-TGCTCCACCTCAGCAAAGTA-3′, gclc sense, 5′-GAGCCATTAGTGCCGTTAGT-3′ and antisense, 5′-G TCTTTCGTCTTCGTCTTGG-3′, gstD1 sense, 5′-TGTACCCTA AGTGCCCCAAG-3′ and antisense, 5′-CTCCAGGAAGGTGT TCAGGA-3′, Rp49 sense, 5′ ACTTCATCCGCCACCAGTC-3′ and antisense, 5′-ATCT CGCCGCAGTAAACG-3′. A 10-fold dilution series of cDNA was used to create the standard curve, and the qRT-PCR efficiency was determined for each gene and each treatment by converting the Ct values into the relative quantities (y = -kx + b). The concentration of the primers in the final reaction was 0.2 μM. The quantity of cDNA is 100 ng and the final volume was adjusted to 20 μL in the final reaction. All results were analyzed by the 2^–ΔΔCt^ method with Rp49 as an internal control.

### Luciferase Assays

The Firefly Luciferase Reporter Gene Assay Kit (Beyotime Biotechnology, Jiangsu, China, RG051S) was used to test luciferase activity. Each sample contained about 15 female midguts. After dissection, they were immediately frozen in liquid nitrogen. Consequently, 50 μL of the Luciferase Reporter Gene Assay Lysis Buffer provided by the manufacturer was added to each sample and then homogenized. Sample extracts were subsequently obtained by centrifugation at 13,000 g at 4°C for 10 min. Luciferase activity was measured following the manufacturer’s instructions.

### Antioxidation Assays

Superoxide dismutase (SOD) activity was measured by Total Superoxide Dismutase Assay Kit with WST-8 (Beyotime Biotechnology, catalog number: S0101S). Catalase activity was measured by Catalase Assay Kit (Beyotime Biotechnology, catalog number: S0051). Malondialdehyde activity was measured by Lipid Peroxidation MDA Assay Kit (Beyotime Biotechnology, catalog number: S0131S). Brief, the main steps include: (1) Sample preparation: Flies were collected at 14, 26, and 40 days post-eclosion. The dissected guts were collected, and the tissue homogenate was prepared. (2) Total protein assay: Total protein of each sample was measured by BCA Protein Assay Kit (Beyotime Biotechnology, catalog number: P0012S). (3) Measuring the SOD, CAT, and MDA activities following the instructions from the manufacturers, respectively. (4) Data analysis: calculating the SOD, CAT, and MDA activities according to the standard curve, respectively.

### Bromophenol Blue Assay

The bromophenol blue assay was performed as previously described to reveal the pH change in the midguts (Li et al., 2016). One hundred microliters of 2% Bromophenol blue sodium (Sigma, B5525) were added to the food surface. Then, several holes were poked to allow the Bromophenol blue solution full absorption. Twelve hours later, images were captured after dissection.

### Cafe Assay and Fly Excretion Measurements

The Cafe and fly excretion measurements were performed as previously described (Cognigni et al., 2011; Deshpande et al., 2014). Briefly, two capillaries (53432-706, VWR) containing 5 μL of liquid food were used. Then, food consumption-ability was calculated from volume reduction.

### Caffeic Acid Feeding Assay

Caffeic Acid (CA) (Aladdin, C108307) was dissolved in DMSO and then added to a standard food medium. Female flies not older than 3 days post-eclosion were collected and distributed equally into food vials containing CA mixed food. An equal volume of DMSO was added to control food.

### “Smurf” Assay

All the flies used were maintained on standard medium until the Smurf assay was performed. The dyed medium was prepared using standard media with Blue dye no. 1 (FD and C Blue No. 1, Spectrum Chemical Manufacturing Corp., FD110) added at a concentration of 2.5% (wt/vol). Flies were kept on the dyed medium for 9 h, followed by counting. A fly was counted as a Smurf when the dye coloration could be observed outside the digestive tract (Rera et al., 2011, 2012).

### Lifespan Assays Under Normal and Stressful Conditions

One hundred females (1–2 days old) of *W*^1118^ were collected and randomly allocated to the CA medium or regular food medium. These flies were transferred to a fresh food source three times per week, during which any deaths and censors were recorded every 2 days. The survival curves were illustrated.

For survival tests under stress conditions, flies were divided into four groups: the control, CA (0.2 mg/mL), paraquat (10 mM), and the paraquat supplemented with CA groups. For each group, 100 female flies were collected and distributed equally into four vials. Simultaneously, 10 males (1–2 days old) were added to each vial to ensure that the females were mated. Female flies that were still alive were counted daily.

### Quantification and Statistical Analysis

All statistical data are presented as the means ± standard deviation (SD) from at least three independent experiments. One-way ANOVA test was used to determine statistical significance unless otherwise mentioned. *P*<0.05 was considered statistically significant. GraphPad Prism version 8.0 was used to evaluate statistical significance after verifying the normality and equivalence of variances.

## Data Availability Statement

The raw data supporting the conclusions of this article will be made available by the authors, without undue reservation.

## Author Contributions

XS: conceptualization, investigation, and writing-original draft. XS and GD: methodology. XS, HC, JZ, YZ, LY, and ZL: writing-review and editing. HC: funding acquisition, resources, and supervision. All authors contributed to the article and approved the submitted version.

## Conflict of Interest

The authors declare that the research was conducted in the absence of any commercial or financial relationships that could be construed as a potential conflict of interest.

## Publisher’s Note

All claims expressed in this article are solely those of the authors and do not necessarily represent those of their affiliated organizations, or those of the publisher, the editors and the reviewers. Any product that may be evaluated in this article, or claim that may be made by its manufacturer, is not guaranteed or endorsed by the publisher.

## Supplementary Material

Supplementary Figure 1 Inhibitory effects of CA on the age-related decline in intestinal functions, related to Figure 2. **(A)** Quantification of GI tract of *Drosophila* fed with the pH indicator Bromophenol Blue. *N* = 90 flies per group. The *P*-values between 14-day and 40-day are highlighted in the 40-day column. The *P*-values between 40-day and 40-day with 0.02 mg/mL CA supplementation are highlighted in the 40-day with 0.02 mg/mL CA column. The *P*-values between 40-day and 40-day with 1 mg/mL CA supplementation are highlighted in the 40-day with 1 mg/mL CA column. Error bars represent the SD of three independent experiments. **(B)** CAFE assay was used to measure food intake in 14- (the blue line), 40- (the red line), 40-day flies in response to 0.02 mg/mL CA supplementation (the green line), and 40-day flies in response to 1 mg/mL CA supplementation (the yellow line). Error bars represent the SD of three independent experiments. **(C)** Quantification of excretion from flies fed with Bromophenol Blue. Excretions are quantified for 28, 30, 29, and 27 fields in each group of 12 flies. 28, 30, 29, and 27 represent the field and the deposits were counted per field. Error bars represent the SD of three independent experiments. **(D)** Quantification of the percentage of “Smurf” flies of 14-day, 40-day, 40-day with 0.02 mg/mL, and 1 mg/mL CA supplementation after consuming a non-absorbed food dye. Error bars represent the SD of three independent experiments. **(E)** Life span assay. Survival curve (%) of female *W*^1118^ flies treated with (marked with the red curve) or without (marked with the blue curve) 0.02 mg/mL CA starting from the day after eclosion. *N* = 100 flies per group. Three independent experiments were performed. Error bars represent SDs. One-way ANOVA test, Log-rank test for (E). **P* = 0.05, ***P* = 0.01, ****P* = 0.001, *****P* = 0.0001, and non-significant (NS) represents *P* = 0.05.

Supplementary Figure 2 Caffeic acid prevents ISC aging *via* antioxidant ability, related to Figure 5. **(A,B)** Immunofluorescence images of the R4 region of dissected midguts of *Drosophila* carrying *esg^*ts*^-GAL4-*driven *Keap1 RNAi* **(A)**, *Keap1 RNAi* with CA supplementation **(B)**. *esg*-GFP (green; ISC, and EB markers), and Dl (red; ISC marker). Panels **(A,B)** represent merged images and panels **(A’,B’)** are for Dl (red) only. DAPI stained nuclei are shown in blue in panels **(A,B)**. Scale bars represent 25 μm in panels **(A,B)**.

Supplementary Figure 3 Caffeic acid prevents ISC aging by counteracting oxidative stress-associated JNK activity, related to Figure 6. **(A,B)** Immunofluorescence images of the R4 region of dissected midguts of *Drosophila* carrying *esg^*ts*^-GAL4-*driven *UAS-Bsk^*DN*^* **(A)**, *UAS-Bsk^*DN*^* with CA supplementation **(B)**. *esg*-GFP (green; ISC, and EB markers), and Dl (red; ISC marker). Panels **(A,B)** represent merged images and panels **(A’,B’)** are for Dl (red) only. DAPI stained nuclei are shown in blue in panels **(A,B)**. Scale bars represent 25 μm in panels **(A,B)**.

Supplementary Table 1 *Drosophila* lines in this study.

Supplementary Table 2 Reagent table.

## Abbreviations

CA: Caffeic acid
ISC: Intestinal stem cell
GI: Gastrointestinal
EB: Enteroblast
EMC: Enteroendocrine mother cell
EC: Enterocyte
EE: Enteroendocrine cell
JNK: c-JunN-terminal kinase
ROS: Reactive oxygen species
pH3: Phospho-Histone 3
Dl: Delta
PQ: Paraquat
DHE: Dihydroethidium
SOD: Superoxide dismutase
CAT: Catalase
MDA: Malondialdehyde
Bsk: Basket
Hep: Hemipterous
PBS: Phosphate-buffered saline.
