# Supplementary figures and images for: Antioxidant Effects of Caffeic Acid Lead to Protection of Drosophila Intestinal Stem Cell Aging
Source: Front Cell Dev Biol. 2021 Sep 9;9:735483. doi: 10.3389/fcell.2021.735483 (PMC8458758; doi:10.3389/fcell.2021.735483)

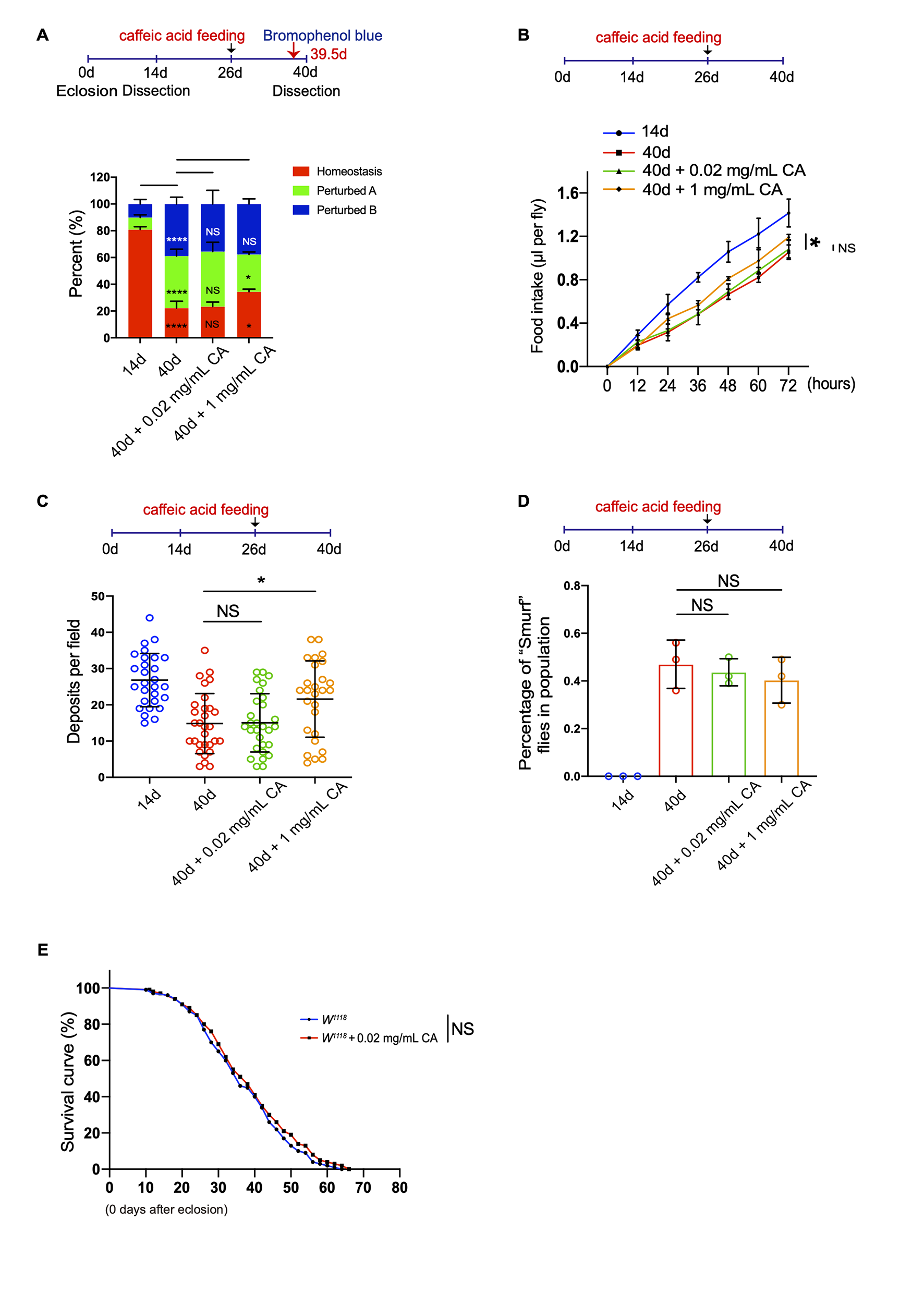

Supplement: Supplementary Figure 1 — Inhibitory effects of CA on the age-related decline in intestinal functions, related to Figure 2. (A) Quantification of GI tract of Drosophila fed with the pH indicator Bromophenol Blue. N = 90 flies per group. The P-values between 14-day and 40-day are highlighted in the 40-day column. The P-values between 40-day and 40-day with 0.02 mg/mL CA supplementation are highlighted in the 40-day with 0.02 mg/mL CA column. The P-values between 40-day and 40-day with 1 mg/mL CA supplementation are highlighted in the 40-day with 1 mg/mL CA column. Error bars represent the SD of three independent experiments. (B) CAFE assay was used to measure food intake in 14- (the blue line), 40- (the red line), 40-day flies in response to 0.02 mg/mL CA supplementation (the green line), and 40-day flies in response to 1 mg/mL CA supplementation (the yellow line). Error bars represent the SD of three independent experiments. (C) Quantification of excretion from flies fed with Bromophenol Blue. Excretions are quantified for 28, 30, 29, and 27 fields in each group of 12 flies. 28, 30, 29, and 27 represent the field and the deposits were counted per field. Error bars represent the SD of three independent experiments. (D) Quantification of the percentage of “Smurf” flies of 14-day, 40-day, 40-day with 0.02 mg/mL, and 1 mg/mL CA supplementation after consuming a non-absorbed food dye. Error bars represent the SD of three independent experiments. (E) Life span assay. Survival curve (%) of female W1118 flies treated with (marked with the red curve) or without (marked with the blue curve) 0.02 mg/mL CA starting from the day after eclosion. N = 100 flies per group. Three independent experiments were performed. Error bars represent SDs. One-way ANOVA test, Log-rank test for (E). *P = 0.05, **P = 0.01, ***P = 0.001, ****P = 0.0001, and non-significant (NS) represents P = 0.05. [file Image_1.TIF]

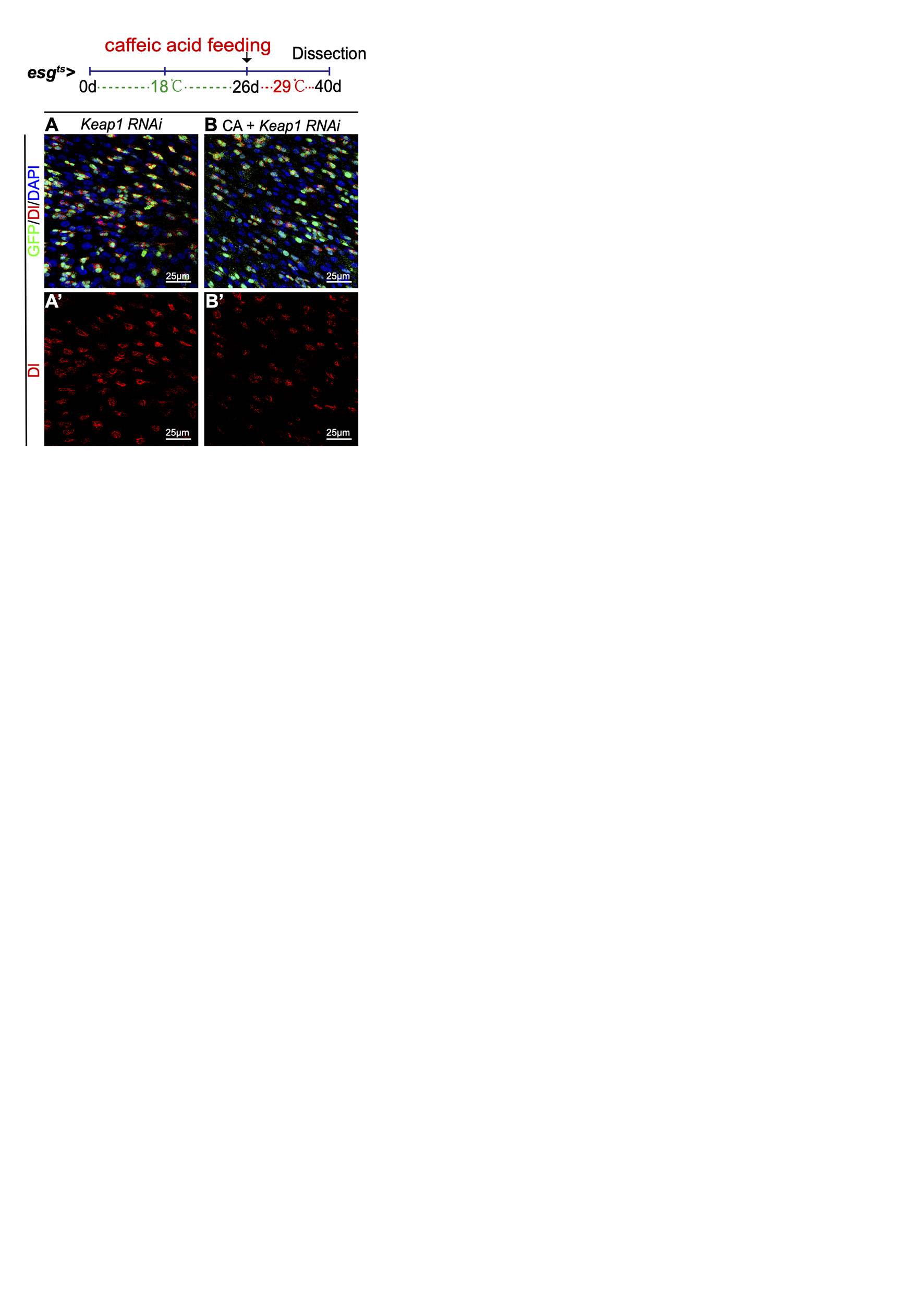

Supplement: Supplementary Figure 2 — Caffeic acid prevents ISC aging via antioxidant ability, related to Figure 5. (A,B) Immunofluorescence images of the R4 region of dissected midguts of Drosophila carrying esgts-GAL4-driven Keap1 RNAi (A), Keap1 RNAi with CA supplementation (B). esg-GFP (green; ISC, and EB markers), and Dl (red; ISC marker). Panels (A,B) represent merged images and panels (A’,B’) are for Dl (red) only. DAPI stained nuclei are shown in blue in panels (A,B). Scale bars represent 25 μm in panels (A,B). [file Image_2.TIF]

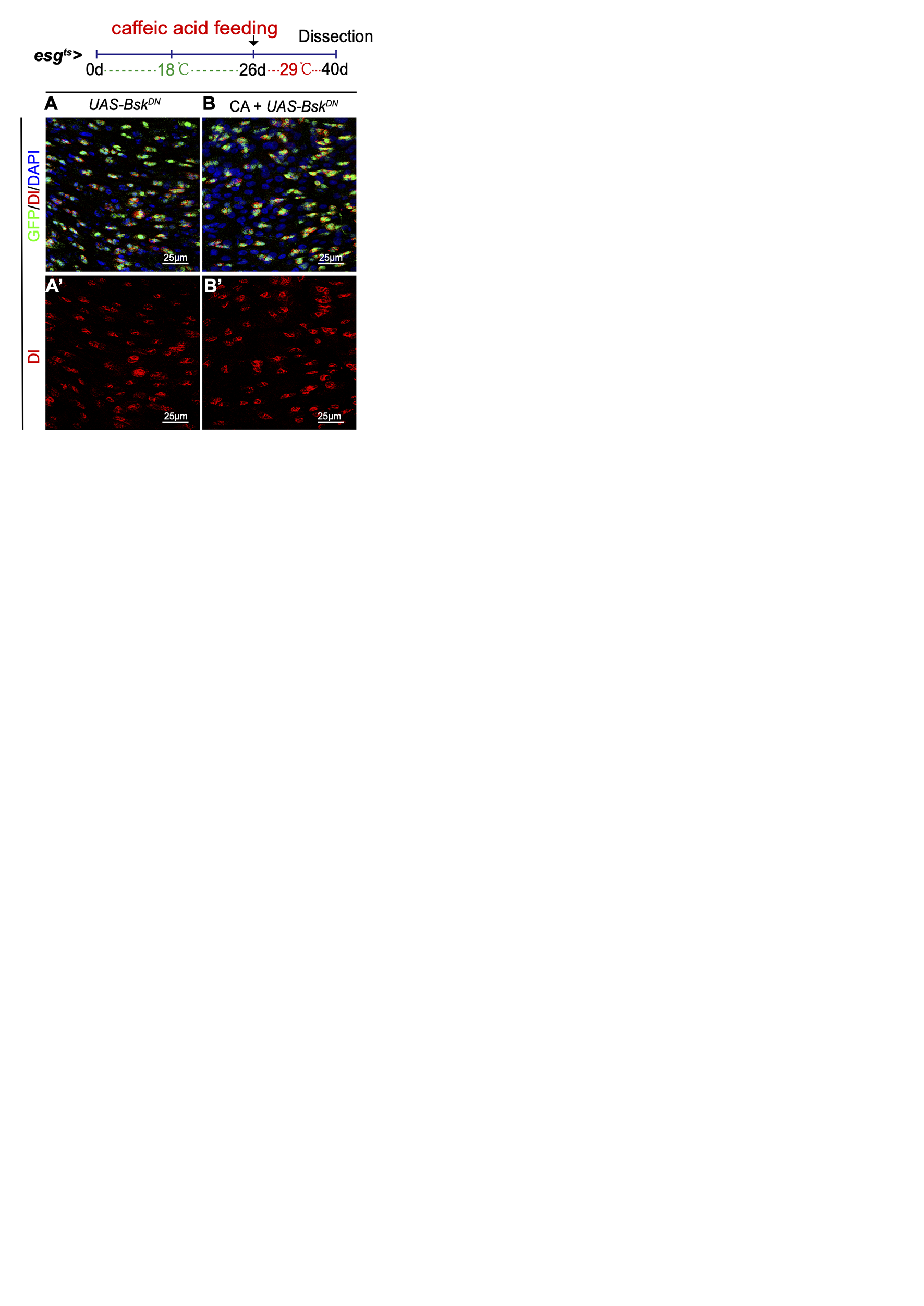

Supplement: Supplementary Figure 3 — Caffeic acid prevents ISC aging by counteracting oxidative stress-associated JNK activity, related to Figure 6. (A,B) Immunofluorescence images of the R4 region of dissected midguts of Drosophila carrying esgts-GAL4-driven UAS-BskDN (A), UAS-BskDN with CA supplementation (B). esg-GFP (green; ISC, and EB markers), and Dl (red; ISC marker). Panels (A,B) represent merged images and panels (A’,B’) are for Dl (red) only. DAPI stained nuclei are shown in blue in panels (A,B). Scale bars represent 25 μm in panels (A,B). [file Image_3.TIF]
